# Diagnostic Difficulties with *Plasmodium knowlesi* Infection in Humans

**DOI:** 10.3201/eid1606.100022

**Published:** 2010-06

**Authors:** Erma Sulistyaningsih, Loeki Enggar Fitri, Thomas Löscher, Nicole Berens-Riha

**Affiliations:** University of Jember, Jember, Indonesia (E. Sulistyaningsih); Brawijaya University, Malang, Indonesia (L.E. Fitr); Universität München, Munich, Germany (E. Sulistyaningsih, T. Löscher; N. Berens-Riha)

**Keywords:** Plasmodium knowlesi, Indonesia, Plasmodium vivax, vector-borne infections, malaria, primates, letter

**To the Editor:** Studies conducted in Malaysia have raised questions about *Plasmodium knowlesi* as the fifth human pathogenic malaria parasite (*1*,*2*); additional cases of *P. knowlesi* malaria have subsequently been reported from other Asian countries (*3*–*5*). Microscopic diagnosis is hindered because *P. knowlesi* morphologically resembles *P. falciparum* or *P. malariae*, depending on blood stage (*6*). Singh et al. has designed a nested PCR assay for identification of *P. knowlesi* infections (*1*).

As part of an ongoing research project focusing on characterizing genes from malaria isolates in Indonesia (E. Sulistyanisih, unpub. data), during December 2008–February 2009, blood samples from 22 gold miners with uncomplicated malaria were collected in South Kalimantan Province in Indonesia. Ring forms typical for *P. falciparum* were seen during microscopy. DNA was extracted and species were identified by nested PCR by using *Plasmodium* genus- and species-specific primers derived from the small subunit ribosomal RNA gene described elsewhere (*1*). PCR products were directly sequenced and verified by 2 independent amplifications of the same DNA sample. PCR using *P. knowlesi*–specific primers yielded a 153-bp product in samples from 4 of the 22 malaria cases. Sequencing showed perfect matching with the recently published *P. knowlesi* S-type from Malaysian Borneo for 1 of the 4 samples. The other sequences were repeatedly consistent with the small subunit ribosomal RNA gene of sporozoite *P. vivax* (S-type), and random blasting (http://blast.ncbi.nlm.nih.gov) showed higher homology (93%–100%) with various *P. vivax* strains than with different *P. knowlesi* (<84%) or other *Plasmodium* strains. The vivax-specific PCR showed the expected bands in each case, and sequencing confirmed *P. vivax* A-type DNA that matched perfectly with a strain from Thailand. Of the miners with malaria, 3 case-patients were coinfected with *P. falciparum*. All 22 samples from the case-patients were negative for *P. malariae*. One case-patient (P 15) infected with *P. knowlesi* (4,000 parasite ring forms/μL) had a mixed infection with *P. vivax* and was successfully treated with chloroquine-primaquine (Table).

**Table:** Profile of *Plasmodium knowlesi*–positive patients, South Kalimantan Province, Indonesia, December 2008–February 2009*

Patient no.	Age, y	Microscopy-based diagnosis	PCR-based diagnosis	*P. knowlesi*–specific PCR for quality of 153-bp band	Sequence analysis of 153-bp sequence
8	35	*P. falciparum*	*P. falciparum, P. vivax, P. knowlesi*	Strong	*P. vivax*
9	41	*P. falciparum*	*P. falciparum, P. vivax, P. knowlesi*	Strong	*P. vivax*
14	54	*P. falciparum*	*P. falciparum, P. vivax, P. knowlesi*	Weak	*P. vivax*
15	16	*P. falciparum*	*P. knowlesi, P. vivax*	Weak	*P. knowlesi* (GU233448)†

*All patients were men who received a diagnosis of uncomplicated malaria. †GenBank accession number.

The results of this study indicate the geographic distribution of natural *P. knowlesi* human infections includes Indonesian Borneo, although this detection is no surprise because many *P. knowlesi* isolates are found in Malaysian Borneo (*1**,**2*)*.* However, the diagnosis would have been unrecognized without molecular techniques, and even those techniques posed a problem.

The species-specific nested PCR assay repeatedly showed bands of 153 bp, indicating 4 *P. knowlesi* cases, but sequencing confirmed *P. knowlesi* in only 1 sample. There was no indication of contamination of the samples tested by PCR, and the other 18 samples and the negative control remained negative for *P. knowlesi*. All 3 samples showed molecularly confirmed mixed infections with *P. falciparum* and *P. vivax* in the case-patients. As *P. vivax* was only molecularly detected, low parasitemia was assumed.

The reverse primer sequence (pmkr 9) is found in *P. vivax* S-type strains and other *Plasmodium* spp., especially those related to *P. vivax*, thus, amplification from this site should be theoretically possible. The forward primer pmk 8, on the other hand, seemed to be highly specific.

One *Plasmodium* strain (GenBank accession no. DQ660817) found in orangutans in Kalimantan, Indonesia, and classified as *P. vivax,* seemed to be more likely to bind to pmk 8 (*7*). However, this classification was recently disproved by Singh and Divis (*8*), and the parasite was categorized as probably being *P. pitheci* or *P. silvaticum*, where human infections are not described. Other primate malaria parasites, such as *P. hylobati*, *P. inui*, *P. cynomolgi*, *P. simium*, *P. fieldi*, and *P. simiovale*, showed better binding sites for pmk 8 than *P. vivax* S- or A-strains. Regarding the theory of *P. vivax* originating in macaques in Southeast Asia and the close relationship to other primate malaria parasites (*9*), one might imagine that *P. vivax* strains in Indonesia differ slightly from the strains described so far. A *P. vivax* isolate from Indonesia, recently sequenced in cooperation with the University of Heidelberg (GenBank accession no. GU233452), showed 2 point mutations; the patient had been in Flores, Bali, and Lembata. However, the 3 *P. vivax* samples presented no mutations at the pmk 8 binding sites. Notably, pmk 8 and pmkr 9 seem always to amplify the S-type and the rVIV 1 and rVIV 2 primers, the A-type DNA, respectively. The genus-specific DNA amplified both types at random.

Some colleagues have experienced similar difficulties with the primers pmk 8 and pmkr 9 in samples from Vietnam (*5*); 2 of 5 samples gave false positive results for *P. knowlesi*. Unfortunately, their report did not mention which species was actually amplified (*5*).

Until recently, we had no satisfying explanation for the 3 assumed false-positive results. Then, in 2009, Imwong et al. reported that these *P. knowlesi* primers stochastically cross-react with *P. vivax* genomic DNA. No polymorphisms alleviating the binding of pmk8 were found; however, a new PCR for *P. knowlesi* was introduced (*10*).

Given the large distribution of the vector and the natural host of *P. knowlesi* in Southeast Asia, it is likely that *P. knowlesi* will be found in other parts of Indonesia. As microscopic and molecular diagnosis of this parasite seems difficult, the underestimation of its distribution and clinical relevance can be assumed.
